# 6-Bromo-4-[(3-chloro-4-methyl­phenyl)­imino­meth­yl]-2-meth­oxy-3-nitro­phenol

**DOI:** 10.1107/S1600536812023859

**Published:** 2012-06-13

**Authors:** Hui Zhu, Hui-Hui Jiang, Hai-Liang Zhu

**Affiliations:** a School of Life Sciences, Shandong University of Technology, Zibo 255000, People’s Republic of China

## Abstract

In the title compound, C_15_H_12_BrClN_2_O_4_, the configuration of the C=N double bond can be described as *trans*. The two aromatic rings in this Schiff base are nearly coplanar with a dihedral angle between their mean planes of 15.4 (2)°. In the crystal, molecules are linked *via* O—H⋯N and C—H⋯O interactions.

## Related literature

For Schiff bases in coordination chemistry, see: Shao *et al.* (2004 ▶) and for their biological activity, see: Desai *et al.* (2001 ▶); Venugopal & Jayashree (2008 ▶). For standard bond lengths, see: Allen *et al.* (1987 ▶). 
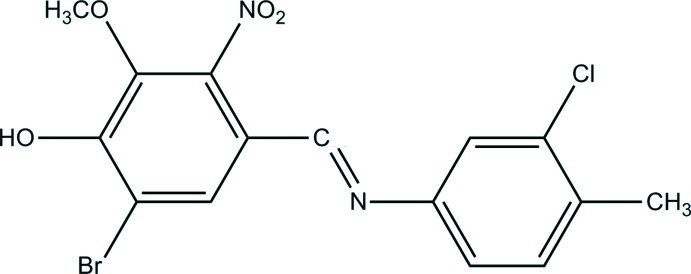


## Experimental

### 

#### Crystal data

C_15_H_12_BrClN_2_O_4_
*M* *_r_* = 399.62
Orthorhombic, 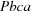

*a* = 18.288 (2) Å
*b* = 8.713 (3) Å
*c* = 19.018 (2) Å
*V* = 3030.4 (11) Å^3^
*Z* = 8
Mo *K*α radiation
μ = 2.91 mm^−1^
*T* = 296 K
0.26 × 0.22 × 0.20 mm

#### Data collection

Bruker SMART APEX CCD diffractometer
Absorption correction: multi-scan (*SADABS*; Bruker, 2000 ▶) *T*_min_ = 0.518, *T*_max_ = 0.594
17176 measured reflections
2973 independent reflections
1713 reflections with *I* > 2σ(*I*)
*R*_int_ = 0.088

#### Refinement

*R*[*F* ^2^ > 2σ(*F* ^2^)] = 0.050
*wR*(*F* ^2^) = 0.149
*S* = 1.06
2973 reflections
210 parameters
H-atom parameters constrained
Δρ_max_ = 0.43 e Å^−3^
Δρ_min_ = −0.49 e Å^−3^

### 

Data collection: *SMART* (Bruker, 2000 ▶); cell refinement: *SAINT* (Bruker, 2000 ▶); data reduction: *SAINT*; program(s) used to solve structure: *SHELXS97* (Sheldrick, 2008 ▶); program(s) used to refine structure: *SHELXL97* (Sheldrick, 2008 ▶); molecular graphics: *SHELXTL* (Sheldrick, 2008 ▶); software used to prepare material for publication: *SHELXTL*.

## Supplementary Material

Crystal structure: contains datablock(s) global, I. DOI: 10.1107/S1600536812023859/vm2167sup1.cif

Structure factors: contains datablock(s) I. DOI: 10.1107/S1600536812023859/vm2167Isup2.hkl

Supplementary material file. DOI: 10.1107/S1600536812023859/vm2167Isup3.cml

Additional supplementary materials:  crystallographic information; 3D view; checkCIF report

## Figures and Tables

**Table 1:** Hydrogen-bond geometry (Å, °)

*D*—H⋯*A*	*D*—H	H⋯*A*	*D*⋯*A*	*D*—H⋯*A*
O2—H2⋯N2^i^	0.82	2.15	2.803 (5)	136
C6—H6⋯O2^ii^	0.93	2.38	3.210 (6)	149
C13—H13⋯O2^ii^	0.93	2.54	3.295 (6)	138

Symmetry codes: (i) 
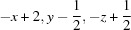
; (ii) 
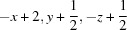
.

**Fig. 1.**
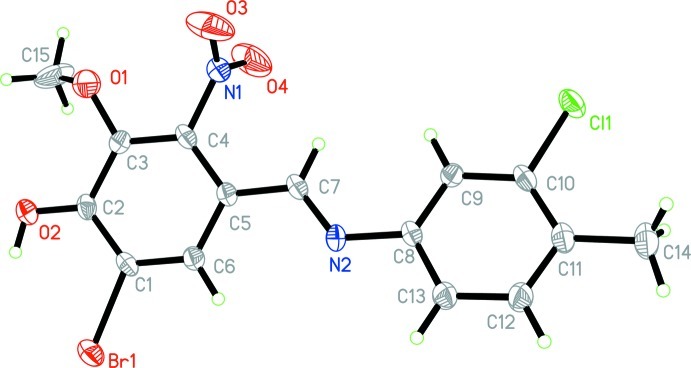
The molecular structure of (I) showing 30% probability displacement ellipsoids.

## supplementary crystallographic information

### Comment

Schiff bases play an important role in the development of coordination chemistry (Shao *et al.*, 2004). Schiff bases have also been shown to exhibit a broad range of biological activities, including antibacterial (Venugopal *et al.*, 2008) and anticancer (Desai *et al.*, 2001) activities. Here we report the synthesis and crystal structure of the title compound (Fig. 1).

The stabilization of the crystal structure is provided by intermolecular hydrogen bonds (Table 1). No π-π interactions are observed in the packing. The compound is weakly twisted, with the dihedral angle between the two benzene rings being 15.4 (2)°. The nitro group makes an angle of 80.7 (3)° withe the best plane through ring C1-C6. All bond lengths are within normal ranges (Allen *et al.*, 1987).

### Experimental

The title compound was synthetized by reaction between 2-bromo-4-hydroxy-3-methoxy-5-nitro-benzaldehyde (2 mmol) and 3-chloro-4 -methylaniline (2 mmol), dissolved in methanol and mixed together for 4 to 5 h. Large block crystals were precipitated, filtered, washed with ethanol and dried in air (yield 80%).

### Refinement

All H atoms were positioned geometrically (O—H = 0.82 Å,

C—H = 0.93–0.96 Å) and were refined as riding, with *U*_iso_(H) = 1.2*U*_eq_(C) or 1.5*U*_eq_(O, methyl C).

### Crystal data

**Table:**

C_15_H_12_BrClN_2_O_4_	*F*(000) = 1600
*M**_r_* = 399.62	*D*_x_ = 1.752 Mg m^−^^3^
Orthorhombic, *P**b**c**a*	Mo *K*α radiation, λ = 0.71073 Å
Hall symbol: -P 2ac 2ab	Cell parameters from 2396 reflections
*a* = 18.288 (2) Å	θ = 2.4–25.2°
*b* = 8.713 (3) Å	µ = 2.91 mm^−^^1^
*c* = 19.018 (2) Å	*T* = 296 K
*V* = 3030.4 (11) Å^3^	Block, orange
*Z* = 8	0.26 × 0.22 × 0.20 mm

### Data collection

**Table:**

Bruker SMART APEX CCD diffractometer	2973 independent reflections
Radiation source: fine-focus sealed tube	1713 reflections with *I* > 2σ(*I*)
Graphite monochromator	*R*_int_ = 0.088
phi and ω scans	θ_max_ = 26.0°, θ_min_ = 2.4°
Absorption correction: multi-scan (*SADABS*; Bruker, 2000)	*h* = −17→22
*T*_min_ = 0.518, *T*_max_ = 0.594	*k* = −10→10
17176 measured reflections	*l* = −23→22

### Refinement

**Table:**

Refinement on *F*^2^	Primary atom site location: structure-invariant direct methods
Least-squares matrix: full	Secondary atom site location: difference Fourier map
*R*[*F*^2^ > 2σ(*F*^2^)] = 0.050	Hydrogen site location: inferred from neighbouring sites
*wR*(*F*^2^) = 0.149	H-atom parameters constrained
*S* = 1.06	*w* = 1/[σ^2^(*F*_o_^2^) + (0.0664*P*)^2^] where *P* = (*F*_o_^2^ + 2*F*_c_^2^)/3
2973 reflections	(Δ/σ)_max_ = 0.001
210 parameters	Δρ_max_ = 0.43 e Å^−^^3^
0 restraints	Δρ_min_ = −0.49 e Å^−^^3^

### Special details

**Table:**

Geometry. All e.s.d.'s (except the e.s.d. in the dihedral angle between two l.s. planes) are estimated using the full covariance matrix. The cell e.s.d.'s are taken into account individually in the estimation of e.s.d.'s in distances, angles and torsion angles; correlations between e.s.d.'s in cell parameters are only used when they are defined by crystal symmetry. An approximate (isotropic) treatment of cell e.s.d.'s is used for estimating e.s.d.'s involving l.s. planes.
Refinement. Refinement of *F*^2^ against ALL reflections. The weighted *R*-factor *wR* and goodness of fit *S* are based on *F*^2^, conventional *R*-factors *R* are based on *F*, with *F* set to zero for negative *F*^2^. The threshold expression of *F*^2^ > σ(*F*^2^) is used only for calculating *R*-factors(gt) *etc*. and is not relevant to the choice of reflections for refinement. *R*-factors based on *F*^2^ are statistically about twice as large as those based on *F*, and *R*- factors based on ALL data will be even larger.

### Fractional atomic coordinates and isotropic or equivalent isotropic displacement parameters (Å^2^)

**Table:**

	*x*	*y*	*z*	*U*_iso_*/*U*_eq_	
Cl1	0.55428 (7)	0.89623 (17)	0.11025 (7)	0.0552 (4)	
Br1	1.09270 (3)	0.59794 (7)	0.22933 (3)	0.0560 (3)	
N1	0.7871 (2)	0.5746 (5)	0.3498 (2)	0.0453 (11)	
N2	0.8252 (2)	0.8332 (4)	0.17064 (18)	0.0337 (9)	
O1	0.8983 (2)	0.4633 (5)	0.4246 (2)	0.0673 (12)	
O2	1.03988 (18)	0.4585 (4)	0.36915 (17)	0.0490 (9)	
H2	1.0785	0.4695	0.3478	0.074*	
O3	0.7666 (3)	0.6579 (6)	0.3947 (3)	0.1000 (18)	
O4	0.7493 (3)	0.4810 (7)	0.3247 (3)	0.0949 (16)	
C1	0.9994 (2)	0.6004 (5)	0.2709 (3)	0.0364 (11)	
C2	0.9893 (2)	0.5226 (5)	0.3358 (2)	0.0346 (11)	
C3	0.9157 (3)	0.5221 (6)	0.3611 (2)	0.0370 (12)	
C4	0.8613 (2)	0.5872 (5)	0.3237 (2)	0.0336 (11)	
C5	0.8729 (3)	0.6696 (5)	0.2597 (2)	0.0311 (10)	
C6	0.9444 (2)	0.6715 (5)	0.2356 (2)	0.0348 (11)	
H6	0.9550	0.7230	0.1940	0.042*	
C7	0.8157 (2)	0.7392 (5)	0.2234 (2)	0.0340 (11)	
H7	0.7681	0.7177	0.2375	0.041*	
C8	0.7679 (2)	0.9012 (5)	0.1301 (2)	0.0321 (11)	
C9	0.6953 (2)	0.8724 (5)	0.1399 (2)	0.0338 (11)	
H9	0.6798	0.8078	0.1758	0.041*	
C10	0.6461 (3)	0.9398 (5)	0.0962 (3)	0.0376 (12)	
C11	0.6652 (3)	1.0380 (6)	0.0428 (3)	0.0446 (13)	
C12	0.7383 (3)	1.0638 (6)	0.0349 (3)	0.0505 (14)	
H12	0.7538	1.1292	−0.0007	0.061*	
C13	0.7898 (3)	0.9972 (6)	0.0774 (3)	0.0434 (13)	
H13	0.8392	1.0172	0.0704	0.052*	
C14	0.6107 (3)	1.1121 (7)	−0.0045 (3)	0.071 (2)	
H14A	0.5802	1.1796	0.0225	0.106*	
H14B	0.5811	1.0346	−0.0263	0.106*	
H14C	0.6358	1.1700	−0.0400	0.106*	
C15	0.9170 (4)	0.3327 (8)	0.4458 (3)	0.083 (2)	
H15A	0.9309	0.2704	0.4064	0.124*	
H15B	0.8767	0.2854	0.4698	0.124*	
H15C	0.9576	0.3423	0.4774	0.124*	

### Atomic displacement parameters (Å^2^)

**Table:**

	*U*^11^	*U*^22^	*U*^33^	*U*^12^	*U*^13^	*U*^23^
Cl1	0.0268 (7)	0.0794 (10)	0.0594 (9)	0.0022 (7)	−0.0050 (6)	−0.0039 (7)
Br1	0.0303 (3)	0.0724 (5)	0.0653 (4)	0.0062 (3)	0.0014 (3)	0.0108 (3)
N1	0.036 (2)	0.057 (3)	0.044 (3)	0.003 (2)	0.000 (2)	0.007 (2)
N2	0.032 (2)	0.035 (2)	0.034 (2)	0.0007 (18)	−0.0099 (17)	−0.0005 (19)
O1	0.053 (3)	0.091 (3)	0.059 (3)	0.021 (2)	0.004 (2)	0.024 (2)
O2	0.0296 (18)	0.070 (2)	0.047 (2)	0.0113 (18)	−0.0033 (16)	0.0053 (18)
O3	0.072 (3)	0.112 (4)	0.116 (4)	−0.001 (3)	0.046 (3)	−0.039 (3)
O4	0.050 (3)	0.112 (4)	0.122 (4)	−0.034 (3)	0.011 (3)	−0.027 (4)
C1	0.022 (2)	0.042 (3)	0.046 (3)	0.000 (2)	−0.002 (2)	−0.005 (2)
C2	0.031 (3)	0.036 (3)	0.036 (3)	0.009 (2)	−0.007 (2)	−0.005 (2)
C3	0.038 (3)	0.044 (3)	0.029 (3)	0.001 (2)	−0.003 (2)	−0.001 (2)
C4	0.024 (2)	0.035 (3)	0.042 (3)	0.004 (2)	−0.003 (2)	−0.009 (2)
C5	0.027 (2)	0.029 (2)	0.037 (3)	0.001 (2)	−0.004 (2)	0.001 (2)
C6	0.030 (3)	0.039 (3)	0.035 (3)	0.003 (2)	−0.002 (2)	0.000 (2)
C7	0.025 (2)	0.036 (3)	0.041 (3)	0.004 (2)	−0.004 (2)	−0.007 (2)
C8	0.026 (2)	0.032 (3)	0.037 (3)	0.004 (2)	−0.004 (2)	−0.004 (2)
C9	0.034 (3)	0.036 (3)	0.031 (2)	0.001 (2)	−0.001 (2)	0.001 (2)
C10	0.025 (2)	0.044 (3)	0.044 (3)	0.003 (2)	−0.005 (2)	−0.009 (2)
C11	0.037 (3)	0.050 (3)	0.047 (3)	0.007 (3)	−0.009 (2)	−0.001 (3)
C12	0.042 (3)	0.053 (4)	0.056 (3)	−0.005 (3)	−0.008 (3)	0.020 (3)
C13	0.036 (3)	0.049 (3)	0.045 (3)	−0.005 (3)	−0.007 (2)	0.005 (3)
C14	0.048 (4)	0.083 (5)	0.080 (5)	0.006 (3)	−0.016 (3)	0.030 (4)
C15	0.111 (6)	0.082 (5)	0.055 (4)	0.013 (4)	0.036 (4)	0.032 (4)

### Geometric parameters (Å, º)

**Table:**

Cl1—C10	1.743 (5)	C6—H6	0.9300
Br1—C1	1.880 (5)	C7—H7	0.9300
N1—O4	1.171 (5)	C8—C9	1.365 (6)
N1—O3	1.182 (6)	C8—C13	1.365 (6)
N1—C4	1.448 (6)	C9—C10	1.358 (6)
N2—C7	1.307 (5)	C9—H9	0.9300
N2—C8	1.429 (5)	C10—C11	1.374 (7)
O1—C15	1.255 (7)	C11—C12	1.362 (7)
O1—C3	1.350 (6)	C11—C14	1.489 (7)
O2—C2	1.253 (5)	C12—C13	1.370 (7)
O2—H2	0.8200	C12—H12	0.9300
C1—C6	1.360 (6)	C13—H13	0.9300
C1—C2	1.420 (7)	C14—H14A	0.9600
C2—C3	1.429 (6)	C14—H14B	0.9600
C3—C4	1.348 (6)	C14—H14C	0.9600
C4—C5	1.429 (6)	C15—H15A	0.9600
C5—C6	1.386 (6)	C15—H15B	0.9600
C5—C7	1.392 (6)	C15—H15C	0.9600
			
O4—N1—O3	122.3 (5)	C13—C8—N2	115.9 (4)
O4—N1—C4	117.9 (5)	C10—C9—C8	118.8 (4)
O3—N1—C4	119.9 (5)	C10—C9—H9	120.6
C7—N2—C8	125.3 (4)	C8—C9—H9	120.6
C15—O1—C3	124.6 (5)	C9—C10—C11	123.6 (5)
C2—O2—H2	109.5	C9—C10—Cl1	116.8 (4)
C6—C1—C2	123.4 (4)	C11—C10—Cl1	119.6 (4)
C6—C1—Br1	118.0 (4)	C12—C11—C10	115.6 (5)
C2—C1—Br1	118.6 (3)	C12—C11—C14	121.2 (5)
O2—C2—C1	123.8 (4)	C10—C11—C14	123.1 (5)
O2—C2—C3	121.6 (4)	C11—C12—C13	122.7 (5)
C1—C2—C3	114.6 (4)	C11—C12—H12	118.7
O1—C3—C4	117.1 (4)	C13—C12—H12	118.7
O1—C3—C2	121.7 (4)	C8—C13—C12	119.4 (5)
C4—C3—C2	121.1 (4)	C8—C13—H13	120.3
C3—C4—C5	123.4 (4)	C12—C13—H13	120.3
C3—C4—N1	118.7 (4)	C11—C14—H14A	109.5
C5—C4—N1	117.9 (4)	C11—C14—H14B	109.5
C6—C5—C7	122.6 (4)	H14A—C14—H14B	109.5
C6—C5—C4	115.4 (4)	C11—C14—H14C	109.5
C7—C5—C4	122.0 (4)	H14A—C14—H14C	109.5
C1—C6—C5	122.0 (4)	H14B—C14—H14C	109.5
C1—C6—H6	119.0	O1—C15—H15A	109.5
C5—C6—H6	119.0	O1—C15—H15B	109.5
N2—C7—C5	123.7 (4)	H15A—C15—H15B	109.5
N2—C7—H7	118.2	O1—C15—H15C	109.5
C5—C7—H7	118.2	H15A—C15—H15C	109.5
C9—C8—C13	119.8 (4)	H15B—C15—H15C	109.5
C9—C8—N2	124.2 (4)		

### Hydrogen-bond geometry (Å, º)

**Table:**

*D*—H···*A*	*D*—H	H···*A*	*D*···*A*	*D*—H···*A*
O2—H2···N2^i^	0.82	2.15	2.803 (5)	136
C6—H6···O2^ii^	0.93	2.38	3.210 (6)	149
C13—H13···O2^ii^	0.93	2.54	3.295 (6)	138

Symmetry codes: (i) −*x*+2, *y*−1/2, −*z*+1/2; (ii) −*x*+2, *y*+1/2, −*z*+1/2.

## References

[bb1] Allen, F. H., Kennard, O., Watson, D. G., Brammer, L., Orpen, A. G. & Taylor, R. (1987). *J. Chem. Soc. Perkin Trans. 2*, pp. S1–19.

[bb2] Bruker (2000). *SMART*, *SAINT* and *SADABS* Bruker AXS Inc., Madison, Wisconsin, USA.

[bb3] Desai, S. B., Desai, P. B. & Desai, K. R. (2001). *Heterocycl. Commun.* **7**, 83–90.

[bb4] Shao, S.-C., You, Z.-L., Fan, S.-H., Tang, L.-L., Xiong, Z.-D. & Zhu, H.-L. (2004). *Acta Cryst.* E**60**, o2183–o2184.

[bb5] Sheldrick, G. M. (2008). *Acta Cryst.* A**64**, 112–122. 10.1107/S0108767307043930 18156677

[bb6] Venugopal, K. N. & Jayashree, B. S. (2008). *Indian J. Pharm. Sci.* **70**, 88–91. 10.4103/0250-474X.40338 PMC2852068 20390087

